# Optical Sensitivity of Waveguides Inscribed in Nanoporous Silicate Framework

**DOI:** 10.3390/nano11010123

**Published:** 2021-01-07

**Authors:** Zhong Lijing, Roman A. Zakoldaev, Maksim M. Sergeev, Andrey B. Petrov, Vadim P. Veiko, Alexander P. Alodjants

**Affiliations:** 1 Faculty of Laser Photonics and Optoelectronics, ITMO University, 197101 Saint Petersburg, Russia; zlj.itmo@gmail.com (Z.L.); mmsergeev@itmo.ru (M.M.S.); 79602507133@yandex.ru (A.B.P.); vpveiko@itmo.ru (V.P.V.); alexander_AP@list.ru (A.P.A.); 2 School of Optical and Electronic Information, Huazhong University of Science & Technology, Luoyu Road 1037, Wuhan 430074, China

**Keywords:** laser direct writing, porous glass, waveguides, photonic circuits, optofluidics, ethanol, small molecules

## Abstract

Laser direct writing technique in glass is a powerful tool for various waveguides’ fabrication that highly develop the element base for designing photonic devices. We apply this technique to fabricate waveguides in porous glass (PG). Nanoporous optical materials for the inscription can elevate the sensing ability of such waveguides to higher standards. The waveguides were fabricated by a single-scan approach with femtosecond laser pulses in the densification mode, which resulted in the formation of a core and cladding. Experimental studies revealed three types of waveguides and quantified the refractive index contrast (up to Δn = 1.2·10^−2^) accompanied with ~1.2 dB/cm insertion losses. The waveguides demonstrated the sensitivity to small objects captured by the nanoporous framework. We noticed that the deposited ethanol molecules (3 µL) on the PG surface influence the waveguide optical properties indicating the penetration of the molecule to its cladding. Continuous monitoring of the output near field intensity distribution allowed us to determine the response time (6 s) of the waveguide buried at 400 µm below the glass surface. We found that the minimum distinguishable change of the refractive index contrast is 2 × 10^−4^. The results obtained pave the way to consider the waveguides inscribed into PG as primary transducers for sensor applications.

## 1. Introduction

A nanoporous silicate framework of porous glass (PG) with multiple buried hollow channels and pores with a well-controlled size in the range of 2–20 nm [1,2] represents a promising matrix, which captures, stores, and transports molecules absorbed from the environment [3,4,5,6,7]. Recently, portable devices with such a PG loaded with organic indicators were demonstrated for monitoring the level of formaldehyde [3], nitrogen dioxide [4], and ozone [5,6] in the environment. The interaction of the indicator and the harmful gases changes the color of the entire glass plate. Laser-induced integration of several activated sectors in the single PG aimed to expand the sensor functionality [8]. However, information processing was performed by the spectral analysis of each sector of the glass plate. For a more accurate analysis of chemical reactions in such a nanoporous medium, it is necessary to inscribe a chip-scale optical channel, namely, a bulk waveguide, which typically consists of a core and cladding to obtain internal reflection of coupled light resulting in a good balance between light localization and optical losses [9]. The optical signal transmitted through the waveguide can trace the media state in real-time, for example, the appearance of new molecules, their chemical interaction, or vice versa evaporation. The combination of a nanoporous silicate matrix and bulk waveguide may provide unique sensing abilities for a nanopore sensor, which operates with single molecules [10,11]. The range of applications depends on the captured molecules and may be demanded in the field of biochemistry [12] or drug investigation [13,14].

Nowadays, femtosecond laser direct writing (LDW) is an advanced technology to fabricate waveguides inside glass materials [15,16,17]. Such structures represent an integral element for photonics circuits [18,19], lab on a chip [20] and sensorics [21]. In particular, various types of optical waveguides have been proposed—gradient [22], core-cladding [23], plasmonic [24,25], and integrated in a glass chip functioning as thermal [26], fluid [21], or light [25] sensors. Most of these applications are based on an evanescent wave interaction with target molecules deposited on a glass surface [21,25,26]. Such a detecting mechanism supposes a waveguide to be incorporated in the glass pre-surface layer to interact with a target fluid deposited on the glass surface. However, that limits 3D integration of waveguides in a photonic chip.

The ability to inscribe waveguides in glass materials enables the development and realization of Mach–Zehnder (MZ) interferometers and quantum photonic circuits possessing low (up to 0.1 dB/cm) dissipation [27,28]. In combination with entangled photon sources of light and efficient single photon detectors, such circuits may be used for linear optics quantum computing [29] and quantum sensorics [30]. In particular, as it is shown in [30], the optofluidic device that contains MZ interferometer and photonic N00N state (with N = 2) at the input, provides the measurement of the concentration of bovine serum albumin (BSA) in aqueous buffer solutions with 10^−3^ accuracy.

Considering all mentioned above, the connection of a waveguide with the sensing medium seems to be relevant. Thus, the concept of waveguides inscription in optically transparent PG can make a significant contribution to the design of optofluidic sensors.

Herein, we suggest a concept of the waveguide in PG to demonstrate a sensing platform for the detection of target molecules. In the first step, we hold the research of waveguides inscription in PG by LDW technique. The investigation revealed three types of waveguides possessing the core-cladding structure. For the sensing application, we utilized the waveguide with the highest refractive index contrast (1.2·10^−2^) and the lowest available propagation losses (~1.2 dB/cm). As a model target molecules, we chose ethanol [31]. Then, we demonstrated that the transmitted laser radiation through the waveguide has a response to ethanol molecules deposited on the PG surface. In particular, the molecules captured by the nanoporous framework transferred to the waveguide cladding and changed the refractive index contrast. This determined and justified the sensitivity of the waveguides to the flow of small objects inside the nanoporous framework. The minimum detectable change in the value of the contrast of the refractive index is 2 × 10^−4^. This indicates the high sensitivity of such a sensor for determining small volumes of molecules. Besides, we showed that the heat treatment of the waveguides did not affect their structural and optical properties, which confirms the multiple uses of the sensor.

## 2. Experiments

### 2.1. LDW of Waveguides in PG

In this work, the femtosecond laser-induced densification approach [32,33] was applied for the inscription of waveguides in PG with an average pore size ~10 nm, and total porosity 26%. Glass composition consists of high silica proportion, SiO_2_ > 96% (mass fraction, %) [34]. These PG plates possess high transparency (~90%) in the visible spectra range, with an average refractive index n ≈ 1.34. The samples were impregnated with water before laser irradiation to expand the ranges of laser processing parameters. Water presence in PG glass provides a more optically homogeneous medium. In [33] we showed that the water impregnation step increases the range of acceptable values of power densities by 15%. The impregnation stage was conducted by immersing the PG sample in distilled water for 96 h at room temperature and atmospheric pressure.

LDW was performed with the linear polarized Gaussian laser beam by Yb-doped fiber laser (Avesta TETA-20, Russia) operating at 1035 nm wavelength with a pulse duration 220 fs, and a fixed repetition rate of 1 MHz. The laser beam was focused by an objective lens (LOMO, 20×, NA = 0.4). The objective lens formed the beam waist with a diameter of 2.5 μm and the Rayleigh length z_0_ = 5.5 μm. The PG samples were mounted on an XYZ translation stage based on a stepper motor with the step equalling 1 µm and being controlled by the driver (SMC-AD3). The waveguides writing was performed by translating the sample perpendicular to the laser beam axis at speed 0.0125–3.75 mm/s and incident pulse energy E_p_ = 0.6, 0.8, and 1.6 µJ. The position of the laser beam focus is 400 μm beneath the upper surface of the glass sample. The length of the writing track was 10 mm.

After the laser writing step, the samples were polished at both facets and heated in a furnace for 2 h at 500 °C, and investigated by optical microscopy (Carl Zeiss, Axio Imager) in the transmission mode and cross-polarized light to show the absence of cracks, stresses, and to study the birefringence of waveguides structure.

### 2.2. Waveguides Testing

**Figure 1:**
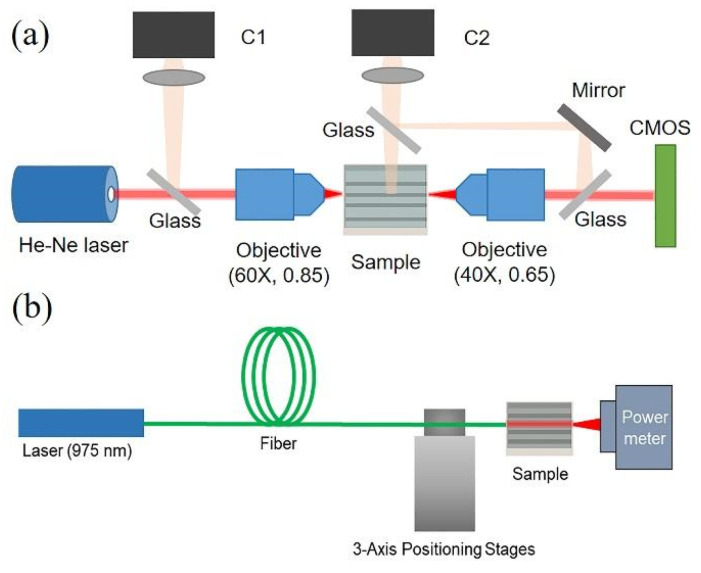
Schemes of the experimental setups for the caption of near-field intensity distribution (**a**) and for measuring losses of waveguides (**b**).

Since the fabricated tracks possess light-guiding properties, they are important to study in terms of the refractive index contrast according to methodology demonstrated by us formerly [35]. For this purpose, a setup equipped with a He-Ne laser and a pair of objectives can register the near-field intensity distribution of radiation transmitted through the waveguide (Figure 1a). In more detail, laser radiation is focused by an objective (60×, 0.85 NA) to couple light into the waveguide. An out-coupled beam is collected by another objective (40×, 0.65 NA). CCD camera (C1) controls the position of the input beam to ensure which part of the waveguide is irradiated. CCD camera (C2) aligns the focus plane of the objective with the out-coupled plane of the waveguide. After that, the near field distribution of the out-coupled beam is captured by CMOS beam profiler (Gentec-EO, Beamage 3.0, QC, Canada). Based on the captured near-field distribution, the refractive index profile of the waveguide is estimated numerically by solving the Helmholtz equation [36]

(1) $$n^{2}(x,y)={\left[n_{b}+\Delta n(x,y)\right]}^{2}=n_{eff}^{2}-\frac{\lambda^{2}}{4\pi^{2}}\frac{\nabla^{2}E(x,y)}{E(x,y)}$$

where, *n_b_* is the refractive index of initial glass, *n_eff_* is the effective refractive index of the propagating mode. Although *n_eff_* is an unknown constant, the refractive index change profile ∆*n*(*x*,*y*) is unaffected by the magnitude of *n_eff_*. With the approximation *n_eff_* = *n_b_*, ∆*n*(*x*,*y*) can be numerically evaluated using *n*^2^(*x*,*y*) ≈ *n_b_*^2^ + 2*n_b_* ∆*n*(*x*,*y*). The normalized E-field distribution *E*(*x*,*y*) across the mode is inferred by the measured near-field intensity of the mode *I*(*x*,*y*). In the calculation, a third-order Butterworth filter with optimized cutoff frequency is employed to remove impulsive and high-frequency noise in the power intensity measured without accuracy loss [37]. The same setup is also utilized in this work for near-field intensity monitoring during ethanol liquid deposition on PG surface (Section 3.4).

The optical losses are also integral parameter, which characterizes the waveguides. The losses measurement are performed with a fiber-coupling setup equipped with a laser module operated at 975 nm, fiber coupling part and power meter (Figure 1b). The input fiber is fixed on high-resolution multi-axis positioning coordinate table with sub-micron accuracy. The Fresnel reflection losses for fiber-to-waveguide connections are considered negligible. The waveguide insertion losses are obtained from the power transmitted through the waveguide and detected with an InGaAs photodiode power sensor (deviation is ±0.5%). The registered signal is normalized to the power propagated through free space with the same distance.

## 3. Results and Discussion

### 3.1. Waveguide Fabrication

**Figure 2:**
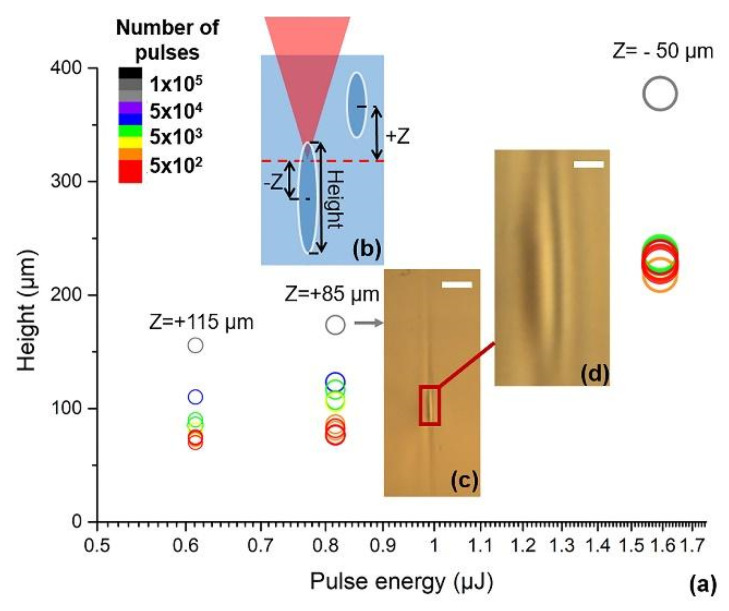
Measured height of waveguides at different pulse energy and number (**a**). Sketch map of laser inscribing and waveguide shift (**b**). The color legend of circles in (**a**) represents the number of laser pulses per region, where a smaller diameter means the maximum positive shift of the waveguide, which is schematically shown in (**b**). Transmission microscope photos of the waveguide fabricated by E_p_ = 0.8 μJ and 10^5^ pulses (**c**) and its enlarged view (**d**). The scale bars are 30 μm (**c**) and 5 μm (**d**).

The pulse energy and scanning speed are two critical parameters determining future waveguide morphology and geometry during the LDW step. The waveguides observed by optical microscopy possess the shape of an elongated ellipse in the cross-section, which is the direct evidence of a filament structure appearance [38]. These filaments are characterized by height in the range of 50–400 µm (Figure 2a) and width 4–7 µm. Such an elliptical shape affects the form of the mode distribution. Another feature is a shift of the waveguide position along the optical axis, depending on the selected pulse energy and the number of laser pulses. The increase of energy and number of laser pulses shifts the waveguide in the positive Z direction, while the decrease causes it to move in the negative Z-direction, as schematically shown in Figure 2b. Moreover, with the high pulses number that is 1.0 × 10^5^, we observed a drop-like structure formed of multiple layers in the cross-section (Figure 2c). The enlarged image shows that the structure of the waveguide consists of a brighter central ellipsoid surrounded by a darker elliptical ring and followed by another slightly brighter ring (Figure 2d). Similar structures were reported in borosilicate glass by Nolte et al. [39] and in Corning Gorilla Glass by Boisvert et al. [40]. The multilayer structure of this kind is interpreted as an occurrence of the densified region and rarefaction region of the glass framework. More details about the waveguide structure are presented in Section 3.3.

### 3.2. Thermal Resistance of Waveguides

PG plate is possible to reuse after capturing analyte and/or reagent by heat treatment in the furnace (temperature up to 500 °C). This reusability is important for the future application of waveguides as a sensing element. However, such a temperature may release the residue stress field of the waveguides or change their properties as a previous study showed for waveguides in solid glass [23]. As part of this work, we confirmed the thermal stability of waveguides when PG with inscribed waveguides is heated in the furnace.

**Figure 3:**
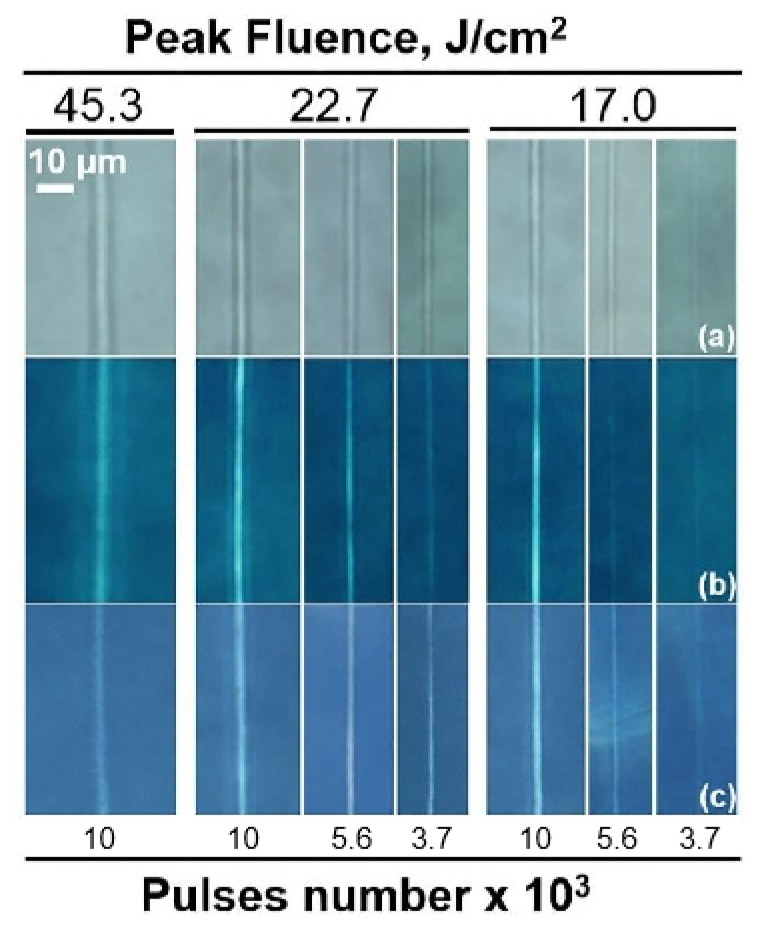
Images of waveguides in transmission light of microscopy (**a**). Polarized microscopy photos of waveguides in top view before (**b**) and after (**c**) heat treatment in a furnace (T = 500 °C, 2 h). The waveguides were fabricated at the peak fluence and number of laser pulses per region, which are indicated in the figure.

Thus, we have tested the thermal stability of waveguides while heating in a furnace at a temperature of 500 °C for 2 h. After the waveguide’s heat treatment, the study was held by transmission microscopy and crossed polarizers to examine birefringent with a constant exposure time (Figure 3). The microscopy in transmission light shows bright elongated regions with the related increased refractive index appeared for peak fluence (F_p_) up to 45 J/cm^2^ and the different number of pulses (Figure 3a). The observed top view in crossed polarizers shows two cases (Figure 3b): bright central part for waveguides fabricated with an increased number of laser pulses (N > 5600) and smooth waveguides (17 J/cm^2^, N < 3700 pulses) whose core does not have a bright color when the sample is rotated relative to crossed polarizers. The bright light is usually associated with birefringence phenomena. However, in comparison with toughened glasses [41], such a glow was found both in the core and in the cladding of the fabricated waveguide. In our study, there is no bright light surrounding the waveguides fabricated with 17.0 < F_p_ < 22.7 J/cm^2^ and pulse number in the range of 3700–5600. That indicates the absence of lateral residual stresses around the waveguides. The surrounded stresses occur for waveguides fabricated by increasing the number of pulses over 10^4^. Even in this case the surrounded stresses relaxed after additional thermal treatment (Figure 3c). The photos of the waveguides’ central part in crossed polarizers after heat treatment had no changes (Figure 3c). This suggests that the internal structure of the waveguides remained the same proving their thermal stability.

### 3.3. Waveguide Properties: Types, Refractive Index Profile, and Losses

**Figure 4:**
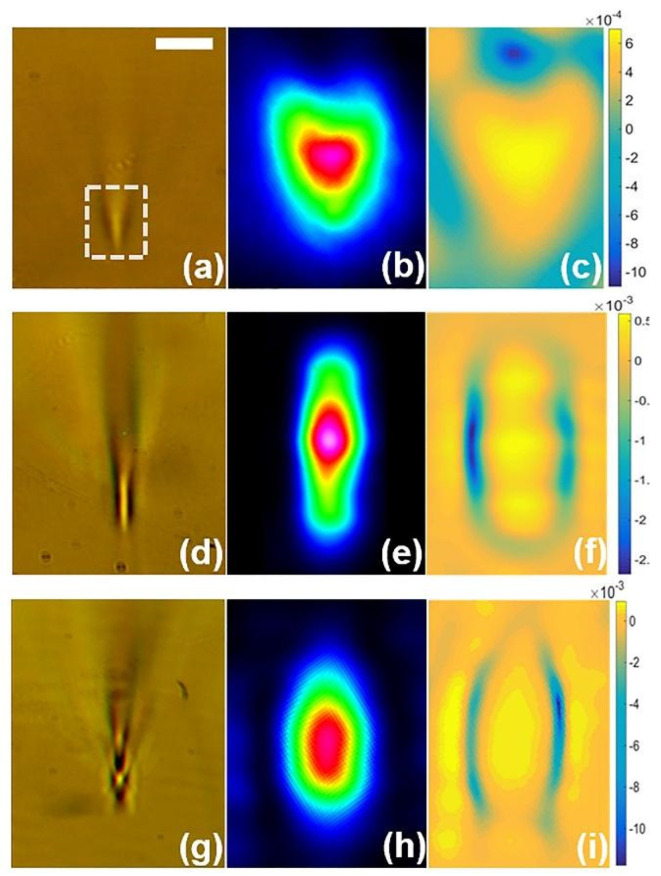
Cross-sectional view of a comet-shaped waveguide (0.6 μJ pulse energy, ~400 pulses) (**a**), rectangular-sectioned waveguide (0.6 μJ pulse energy, ~10^4^ pulses) (**d**) and cylindrical-shaped waveguide (1.6 μJ pulse energy, ~600 pulses) (**g**) with corresponding measured near-field distribution (**b**,**e**,**h**) and estimated refractive index profile (**c**,**f**,**i**). Dash white squares indicate the laser radiation coupling position. Scaling bar equals 10 μm.

The refractive index profile reveals three types of sandwich-like structure of fabricated waveguides. The difference between them is in the reordering of densified and rarefaction layers. The uniform waveguide is written by using relatively low pulse energy 0.6 μJ at a high translating speed of 3.75 mm/s (N ~ 400), with the peak fluence 17.0 J/cm^2^ and net fluence 6.8·10^3^ J/cm^2^. The cross-sectional view of the waveguide shows a comet-like shape with the coupling beam position on the tip (highlighted by a dot-dash outline in Figure 4a). The near-field distribution of propagating laser light through the waveguide is also captured to observe a guiding mode and cross-sectional features (Figure 4b). The near-field distribution exhibits a satisfactory modal profile confirmed by the well-confinement of the guiding light. The refractive index profile evaluated from Equation (1) demonstrates the core (Δn_core_ = 6.5·10^−4^) and cladding (Δn_clad_ = −10.5·10^−4^) (Figure 4c). The refractive index contrast between the core and cladding is 1.7·10^−3^. The shape of the distribution of the refractive index also tends to the shape of a ‘comet’, but the structure remains within the framework of the classical concept of a waveguide. This LDW regime is relatively fast and allows us to fabricate the type of waveguides, which we have called a ‘comet-shaped waveguide’.

With the same pulse energy 0.6 μJ but at the lowest translating speed 0.125 mm/s (N ~ 10^4^) with the same peak fluence but with larger net fluence ~2 × 10^5^ J/cm^2^, an elongated densified region is observed in the cross-sectional view (Figure 4d). That occurs due to the self-focusing of the propagating wavefront, i.e., the pulse collapses into a filament near the focus [42]. The captured near-field image demonstrates a rectangular shape distribution with its maximum in the central part (Figure 4e). The central mode profile is elliptical. The refractive index profile shows an opposing result, where the positive rectangular core (Δn_core_ = 5·10^−4^) is surrounded by symmetrically located negative cladding (Δn_clad_ = −2.7·10^−3^) (Figure 4f). The refractive index contrast between the core and cladding is 3.2·10^−3^. The second type of waveguides are called ‘rectangular-sectioned waveguides’.

The third type of waveguides are written with a relatively high pulse energy of 1.6 μJ at a translating speed of 2.5 mm/s (N ~ 600) with a larger peak fluence of 45.3 J/cm^2^ and net fluence 2.7·10^4^ J/cm^2^. Here, we see a 2-micron-sized cylindrical-shaped core located between two rarefaction layers (Figure 4g). Basically, such a structure occurs as a result of self-focusing and defocusing by self-generated plasma [42]. The coupling of He-Ne laser radiation into the core shows a nearly single elliptical mode distribution and is captured in Figure 4h. Again, the refractive index profile demonstrates a positive elliptic core with maximum Δn_core_ = 6.0·10^−4^ surrounded with a highly depressed cladding with maximum Δn_clad_ = −1.2·10^−2^. The contrast between the core and cladding is 1.2·10^−2^ (Figure 4i). The third type of waveguides are called ‘cylindrical-shaped waveguides’. This type possesses the highest contrast, the absence of residual stresses in the cladding, and is chosen for the following losses and sensing investigations.

**Figure 5:**
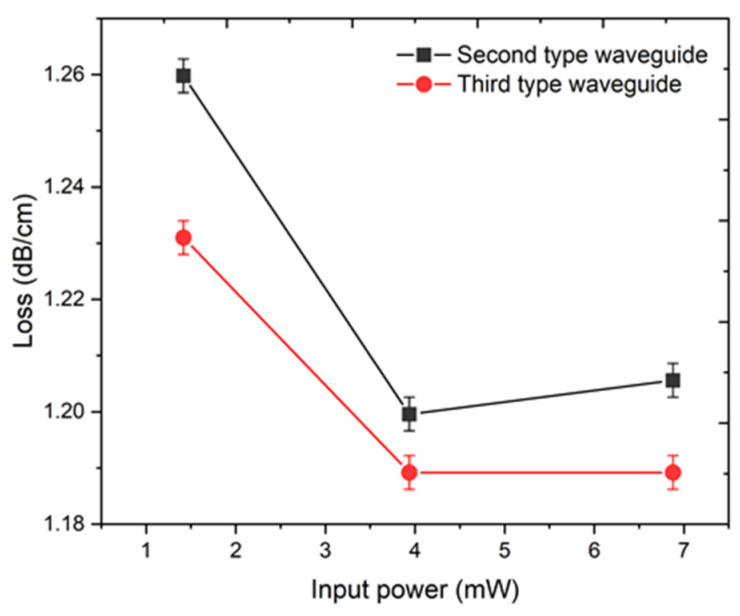
Insertion losses measurement of the second type waveguide (0.6 μJ pulse energy, ~10^4^ pulses), and the third type waveguide (1.6 μJ pulse energy, ~600 pulses).

Insertion losses (α) are measured for the waveguides, which refers to the second type waveguide (Figure 4d–f) and third type waveguide (Figure 4g–i). This was accomplished by coupling laser module radiation into the core of the waveguide on the fiber-coupling setup (Figure 1b). The input power (*P_in_*) was equal to 1.4, 3.9, and 6.9 mW. The output power (*P_out_*) of the light after passing through the waveguide was also registered (Figure 5). The insertion losses are estimated as

(2) $$\alpha =10\cdot \mathrm{lg}\left(P_{in}/P_{out}\right).$$

Thus, the second type waveguide possesses a slightly higher inversion loss compared with the third type waveguide, while the average insertion loss of these waveguides is ~1.2 dB/cm.

Notably, the insertion losses account for both coupling losses and propagation losses. The cross-sectional morphology determines the spatial distribution of the mode supported by the waveguide. Any mismatch between the mode fields of the waveguide and the input beam will result in increased insertion losses. Meanwhile, as in silicate glass [43], the insertion losses originated from the non-symmetrical morphology of the waveguide can be dramatically reduced to below 1 dB/cm at 1550 nm, by optimizing the fabrication process. Moreover, if we compare our result to a waveguide in porous silicon [44], which is applied for sensing application, our waveguides possess an order of magnitude lower losses. Therefore, we can assume that, for a primary transducer with a length of several centimeters, the obtained loss value is suitable.

When the waveguide is used as the analyzer of liquid, the liquid penetrates to the cladding through the porous framework, mitigates the refractive index contrast between the core and cladding, and deteriorates the waveguiding property. Notably, waveguides with a higher refractive index contrast are preferable for liquid sensor applications. Therefore, the third type of waveguides was selected for the sensing demonstration.

### 3.4. Opto-Fluidic Waveguide for Ethanol Molecule Sensing

The occurrence of the optical micro-channel in PG is a remarkable feature, as the fabricated waveguide exhibits sensitivity to the changes happening in the nanoporous framework. Such sensitivity is associated with the waveguide’s cladding filling with molecules deposited on the glass surface. First, these changes can be noticed when the refractive index contrast changes, then there will be a distortion of the intensity distribution at the waveguide output. The key sensor parameter of such a waveguide is a response time.

Here, we demonstrate an approach to detect molecules captured by PG during the registration of time-dependent changes in the near-field mode distribution of the waveguide output. In the experiment we use the ‘cylindrical-shaped waveguide’ due to the following reasons: (i) uniform elliptical mode of distribution; (ii) lowest losses; (iii) the presence of space for liquid precipitation in the form of rarefaction layers around the waveguide core. To conduct this study, widely available ethanol, a so-called “small molecule” [45], was chosen as the liquid, since the molecules easily penetrate through PG nanoporous framework [46].

**Figure 6:**
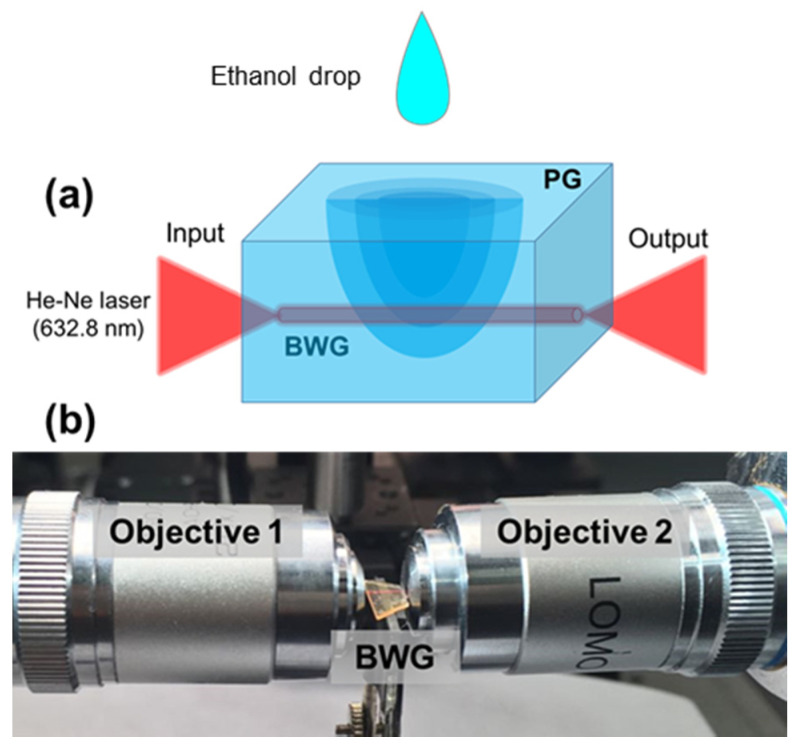
(**a**) Schematic view of liquid-immersing procedure for PG with the inscribed cylindrical-shaped waveguide. (**b**) Photo of the experimental setup for waveguide sensor ability testing.

The experimental scheme for waveguide testing is given in Figure 6. He-Ne laser radiation is coupled into the waveguide. An objective-objective connection is applied to laser radiation coupling and registering the transmitted laser radiation through the waveguide (Figure 6a). The objectives are fixed on the multiple-axis translation stages to provide accurate positioning of both the in-coupling beam and out-coupling beam (Figure 6b). A CMOS camera located behind objective 2 captures the near-field intensity distribution, as schematically shown in Figure 1a.

**Figure 7:**
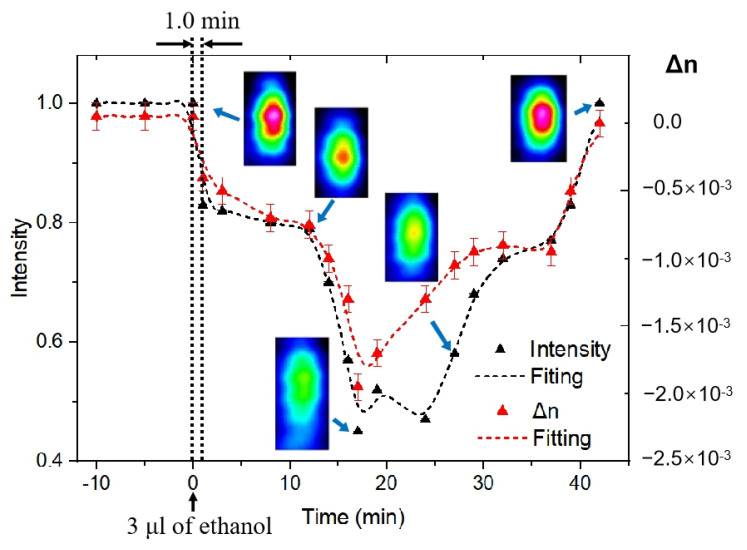
Time dependence of the peak intensity of near-field distribution (black triangle) and the change of the refractive index contrast between core and cladding of waveguides (red triangle) after PG impregnation with 3-μL ethanol liquid.

In the experiment, a 3 μL-drop of ethanol liquid was deposited on the PG surface, where the monitoring of near-field intensity distribution at the waveguide output occurred during 45 min after ethanol deposition (Figure 7). The exposure time of the CMOS camera was constant (20 ms) to trace the intensity change of the guiding light. The RMS noise of the CMOS camera is 1000:1. In the first minute of ethanol penetration, there was a sharp jump in the value of the transmitted intensity, the same was observed in the value of the contrast (red dots/curve in Figure 7). After 15 min, the mode shape was changed with the corresponding intensity decrease. Meanwhile, the filling of nanopores with ethanol mitigates the refractive index contrast between the core and cladding of the waveguide, and thus deteriorates its light guiding ability. After 17 min, we noticed the maximum decrease in the intensity, the added ethanol diffused to the region adjacent to the waveguide through connected nanopores of PG. 22 min later, the intensity began to recover, reaching its original state after 42 min. The results obtained show the waveguide possesses an ability to detect small molecules such as ethanol captured by PG upon registration of time-dependent changes in the near-field intensity distribution at the waveguide output.

**Figure 8:**
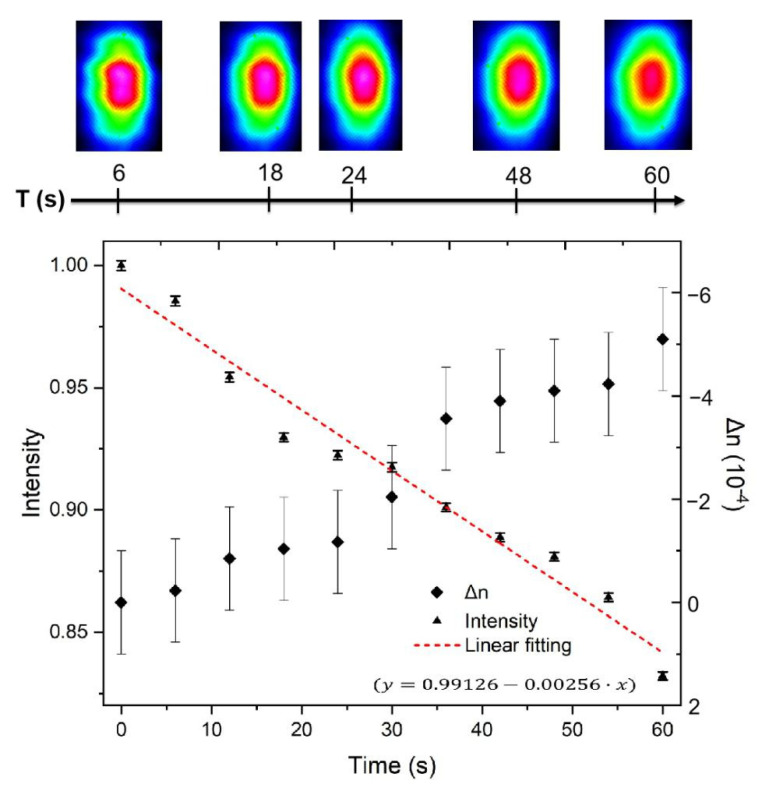
Evolution of the peak intensity of the near-field distribution and the change of refractive index contrast during the first minute after ethanol deposition on the PG surface. The inserted images are the near-field distributions at 6, 18, 24, 48, and 60 s.

To estimate the response time of the waveguide, we captured the evolution of the near-field intensity distribution during the first minute after the ethanol deposition with an interval of 6 s (that corresponds to the minimum measurement interval of CMOS camera) and a constant exposure time of 20 ms. The result is given in Figure 8, where an approximately linear decline is revealed with a slope of 0.00248 s^−1^. The time of ethanol liquid deposition on the PG surface was set as the beginning of the recording. There is a significant decrease in the intensity of about 17% within 60 s, which can be easily distinguished by most photodiode detectors (for example, Hamamatsu Photonics S5972 PIN Photodiode). Applying our CMOS camera parameters, we can establish the response time is fewer than 6 s. Meanwhile, our numerical simulation of the refractive index profile reveals a decrease of refractive index contrast of ~5 × 10^−4^ with a deviation error of ±1 × 10^−4^ within 60 s. Therefore, the minimum distinguishable change in the value of the refractive index contrast is ~2 × 10^−4^.

## 4. Conclusions

In conclusion, we have applied LDW in PG to fabricate new types of waveguides. Specifically, a gradual sequence of uniform ‘comet-shaped’, ‘rectangular-sectioned’, and ‘cylindrical-shaped’ waveguides was achieved in PG by varying pulse energy and/or the number of accumulated laser pulses. The ‘cylindrical-shaped’ waveguide is written by the following parameters: 1 MHz, 1.6 μJ pulse energy, and 2.5 mm/s translating speed, which is characterized by relatively high ∆n ~1.2·10^−2^. The obtained value is suitable for the current tasks of the sensor element. For other applications this value can be increased by adopting the multi-scan technique [47]. However, the detailed investigation of the level of nanopore collapse in the waveguide cross-section is required. Waveguide optical losses represent one of important issues for various applications in photonics and quantum technologies. For the currently available LDW setup, the insertion losses of fabricated ‘cylindrical-shaped waveguide’ are equal to 1.2 dB/cm. The obtained level of insertion losses is due to the technical imperfections of the experimental setup. The losses can be significantly reduced by implementing with the currently available technology utilized the position accuracy of hundreds of nanometers [48].

In particular, the ‘cylindrical-shaped’ waveguide is used for the detection of ethanol molecules deposited on PG upon registration of time-dependent changes in the near-field distribution at the waveguide output. The waveguide shows a short response time of ~6 s and high sensitivity with a minimum detectable change in the value of the refractive index contrast of the numerical method of ~2 × 10^−4^. The results show the optical sensitivity of waveguides inscribed in PG for the detection of small molecules such as ethanol. Then, on the next step, the calibration procedure of such a sensor is required for every target molecule. Depending on the properties of the molecules, the calibration can be accomplished by the response time, minimum/maximum intensity level shift at the waveguide output, and the recovery time. The size of pores places a restriction on the molecule to be detected—molecules larger than the pore size cannot be detected because they are unable to penetrate the waveguide cladding.

It is worth noticing that our recent work enables to use PG waveguides for applications in the quantum domain even in the presence of some moderate level of losses. In particular, further improvement of the considered technology allows to design MZ interferometers as a basis for high precision sensors and photonic information processing circuits [49]. For sensor applications, one can use the waveguide in an interferometer arm to obtain an interference pattern to show the concentration variation of captured molecules. The entangled states and especially, N00N states at the input of the interferometer can improve the visibility for this method [30].

## Data Availability

Data sharing not applicable.
